# COVID-19 vaccine-related adverse events following immunization in the WHO Western Pacific Region, 2021–2022

**DOI:** 10.5365/wpsar.2023.14.2.1046

**Published:** 2023-06-24

**Authors:** Ananda Amarasinghe, Heeyoun Cho, Eve Rizza Katalbas, Yoshihiro Takashima

**Affiliations:** Vaccine-Preventable Diseases and Immunization, Division of Programs for Disease Control, World Health Organization Regional Office for the Western Pacific, Manila, Philippines.

## Abstract

The speed at which new vaccines against coronavirus disease (COVID-19) were developed and rolled out as part of the global response to the pandemic was unprecedented. This report summarizes COVID-19 vaccine-related safety data in the World Health Organization Western Pacific Region. Data for 1 March 2021 to 31 March 2022 from 36 out of 37 countries and areas in the Western Pacific Region are presented. More than 732 million doses of eight COVID-19 vaccines were administered; reporting rates of adverse events following immunization (AEFIs) and serious AEFIs were 130.1 and 5.6 per 100 000 doses administered, respectively. Anaphylaxis, thrombosis with thrombocytopenia syndrome, and myocarditis/pericarditis were the most frequent COVID-19 adverse events of special interest (AESIs) reported. The reported rates of AESIs in the Western Pacific Region were within the range of expected or background rates. Vaccine benefits far outweigh the risk of reported serious adverse reactions and serious outcomes of COVID-19. Continued AEFI surveillance is recommended to better understand and ensure the safety profiles of novel COVID-19 vaccines.

The global coronavirus disease (COVID-19) immunization campaign was unprecedented in its scale, speed and specificities. (*1*, *2*) The first COVID-19 vaccine was granted emergency use listing (EUL) by the World Health Organization (WHO) in December 2020. (*3*) By 31 March 2022, nine COVID-19 vaccines had received EUL, (*4*) eight of which have been used in the WHO Western Pacific Region.

While the development and EUL approval processes of COVID-19 vaccines were accelerated, the quality and safety of COVID-19 vaccines were not compromised, as evidenced by the clinical trials conducted in the development phase (*5*) and the robust vaccine and immunization safety monitoring mechanisms that were established post-licensure. The latter are an essential part of ensuring the safety of vaccines and were especially important in the case of COVID-19 given the large target population (which included different age groups and high-risk individuals) and the simultaneous use of different COVID-19 vaccines.

Many countries expanded their existing surveillance systems for adverse events following immunization (AEFIs) to include the monitoring of COVID-19 vaccine safety events. Data collected by these systems on AEFIs with COVID-19 vaccines were routinely reported to WHO. This paper reviews the available surveillance data on COVID-19 vaccine-related AEFIs from countries and areas in the Western Pacific Region during 1 March 2021–31 March 2022.

## Methods

### Definitions

An AEFI is defined as any untoward medical occurrence that follows immunization; AEFIs do not necessarily have a causal relationship with the use of a vaccine. A serious AEFI is defined as an event that is life-threatening or results in inpatient hospitalization or prolongation of existing hospitalization, persistent or significant disability/incapacity, a congenital anomaly/birth defect or death. (*6*) Adverse events of special interest (AESIs) are a subset of AEFIs and are defined as a pre-specified medically significant condition that has the potential to be causally associated with a vaccine product and that needs to be carefully monitored and/or confirmed by further studies. (*7*)

### Data sources

Data were obtained from 36 of the 37 countries and areas in the WHO Western Pacific Region; no data were available from China (**Table 1**). COVID-19 vaccination and safety data for the period, 1 March 2021 to 31 March 2022, were collated from weekly reports provided by WHO country offices and countries; for countries that did not provide weekly reports, safety data were obtained from publicly available data published on official government web sites (e.g. web sites of ministries or departments of health or national regulatory agencies). For some countries and areas, both weekly reports and data from official government web sites were used (**Table 1**). Inconsistent and incomplete data were followed up with the corresponding WHO country offices or government focal points for COVID-19 vaccine data.

**Table 1:** Sources of COVID-19 safety data, by country and area in the Western Pacific Region

Data source	Country and area^a^
**Weekly reports**	Non-PICs: Brunei Darussalam, Cambodia, Hong Kong Special Administrative Region SAR (China), Lao People's Democratic Republic, Macao SAR (China), Malaysia,^b^ Mongolia, New Zealand,^b^ Papua New Guinea, the Philippines, Viet Nam
	PICs: American Samoa, Cook Islands, Fiji,^b^ Guam, Kiribati, Marshall Islands, Federated States of Micronesia, Nauru, Niue, Commonwealth of the Northern Mariana Islands, Palau, Pitcairn Islands, French Polynesia,^b^ Samoa, Solomon Islands, Tokelau, Tonga, Tuvalu, Vanuatu, Wallis and Futuna
**Official government web sites**	Non-PICs: Australia, Japan, Malaysia,^b^ New Zealand, Republic of Korea, Singapore
	PICs: New Caledonia, Fiji,^b^ French Polynesia^b^

PICs: Pacific island countries and areas; SAR: Special Administrative Region.

^a^ There are 27 countries and 10 areas in the Western Pacific Region.

^b^ Safety data were obtained from both weekly reports and data published on official government web sites.

Data reported through the Regional event-based surveillance (EBS) (*8*) system were used to supplement the analysis. The EBS system was established as an early warning mechanism to rapidly capture publicly reported safety events related to COVID-19 vaccination, including AESIs reported by regional and global sources such as media International Health Regulations (2005) reports, and government agency reports and publications. It was established by, and functions with, the guidance of the Health Emergencies Programme team at the WHO Regional Office for the Western Pacific.

Four categories of AESIs are included in this report: anaphylaxis, thrombosis with thrombocytopenia syndrome (TTS), myocarditis/pericarditis and Guillain-Barré syndrome (GBS). Although WHO’s COVID-19 safety surveillance manual defines anaphylaxis as a severe immediate (within 1 hour) allergic reaction leading to circulatory failure with or without bronchospasm and/or laryngospasm/laryngeal oedema, (*7*) the case definitions and diagnostic criteria (*9*) used by countries for anaphylaxis varied across the Region.

TTS was defined as the presence of a thrombosis/thromboembolism, generally in uncommon anatomical locations (such as cerebral venous sinus or splanchnic veins) and marked thrombocytopenia following vaccination with a COVID-19 non-replicant adenovirus vector-based vaccine. (*10*) At the start of 2021, the detection and reporting of TTS was compromised by uncertainty in the pathogenesis, complicated clinical and laboratory presentations and the lack of a clear case definition for TTS. However, TTS surveillance quickly improved as new evidence became available and guidelines evolved during May and June 2021. Only rates of TTS following immunization with the Vaxzevria (AstraZeneca) COVID-19 vaccine were reported; despite reports of TTS following administration of the Ad26.COV2.S (Janssen or Johnson & Johnson) COVID-19 non-replicant adenovirus vector-based vaccine globally, and although 14 countries and areas in the Region had introduced this vaccine, disaggregated data were not available to assess TTS rates for the Johnson & Johnson vaccine in the Western Pacific Region.

Myocarditis is an inflammation of the heart muscle, and pericarditis is an inflammation of the lining that surrounds the heart. In July 2021, the COVID-19 subcommittee of the WHO Global Advisory Committee on Vaccine Safety (GACVS) issued a statement regarding reports of myocarditis and pericarditis following administration of COVID-19 mRNA vaccines and encouraged reporting of these two conditions. (*11*)

GBS is a rare, serious neurological autoimmune disorder that affects the peripheral nervous system and can lead to weakness and paralysis. GBS has been observed following some viral and bacterial infections and, more rarely, following the use of some vaccines including influenza vaccines. (*12*) In July 2021, the WHO GACVS COVID-19 subcommittee issued a statement regarding reports of GBS following administration of adenovirus vector-based COVID-19 vaccines. (*13*)

### Data analysis

Data were used to calculate rates of reported AEFIs and AESIs (anaphylaxis, TTS, myocarditis and/or pericarditis and GBS) per 1 million doses administered. Reporting rates were calculated separately for Pacific island countries and areas (PICs) and non-PICs. Where either the numerator (number of adverse events) or the denominator (number of administered doses) was not available separately, i.e. disaggregated by vaccine, these data were excluded from the computation of AEFI rates.

## Results

### Vaccines used in the Western Pacific Region

Between 1 March 2021 and 31 March 2022, more than 732 million doses of the seven WHO EUL-granted  COVID-19 vaccines – Comirnaty (Pfizer-BioNTech), Spikevax (Moderna), AstraZeneca, Johnson & Johnson, BBIBP-CorV (Sinopharm), CoronaVac (Sinovac) and Nuvaxovid (Novavax) – and one non-WHO EUL COVID-19 vaccine, Gam-Covid-Vac (Gamaleya) – were administered across 36 countries and areas in the Region. The most widely used vaccine was the Pfizer-BioNTech vaccine (433.7 million doses administered in 29 countries and areas), followed by the Moderna vaccine (101.8 million doses administered in 17 countries and areas). Although the Sinovac vaccine was administered in relatively few countries in the Region, it ranked third in terms of number of doses administered (**Table 2**).

**Table 2:** COVID-19 vaccine introductions during 1 March 2021–31 March 2022, by country and area in the Western Pacific Region

Country and area	Vaccine							
	Pfizer-BioNTech BNT162b2	Moderna mRNA-1273	Sinovac	AstraZeneca-Oxford University AZD1222	Sinopharm COVID-19 vaccine BIBP	Johnson & Johnson Janssen Ad26.COV2.S	Gamaleya Gam-COVID-Vac^a^	Novavax NVX-CoV2373
**Non-PICs**								
Australia	Y	Y	N	Y	N	N	N	Y
Brunei Darussalam	Y	Y	N	Y	Y	N	N	N
Cambodia	Y	Y	Y	Y	Y	Y	N	N
Hong Kong Special Administrative Region SAR (China)	Y	N	Y	N	N	N	N	N
Japan	Y	Y	N	Y	N	N	N	N
Lao People's Democratic Republic	Y	N	Y	Y	Y	Y	Y	N
Macao SAR (China)	N	N	N	N	Y	N	N	N
Malaysia	Y	N	Y	Y	Y	N	N	N
Mongolia	Y	N	N	Y	Y	N	Y	N
New Zealand	Y	N	N	Y	N	N	N	Y
Papua New Guinea	N	N	N	Y	Y	Y	N	N
Philippines	Y	Y	Y	Y	Y	Y	Y	N
Republic of Korea	Y	Y	N	Y	N	Y	N	Y
Singapore	Y	Y	Y	N	Y	N	N	N
Viet Nam	Y	Y	N	Y	Y	N	Y	N
**PICs**								
American Samoa	Y	Y	N	N	N	Y	N	N
Cook Islands	Y	N	N	N	N	N	N	N
Fiji	Y	Y	N	Y	N	N	N	N
French Polynesia	Y	N	N	N	N	Y	N	N
Guam	Y	Y	N	N	N	Y	N	N
Kiribati	N	N	N	Y	Y	N	N	N
Marshall Islands	Y	Y	N	N	N	Y	N	N
Federated States of Micronesia, Federated States of	Y	Y	N	N	N	Y	N	N
Nauru	Y	N	N	Y	N	N	N	N
New Caledonia	Y	N	N	N	N	Y	N	N
Niue	Y	N	N	N	N	N	N	N
Northern Mariana Islands, Commonwealth of the	Y	Y	N	N	N	Y	N	N
Palau	Y	Y	N	N	N	Y	N	N
Pitcairn Islands	N	Y	N	Y	N	N	N	N
Samoa	Y	N	N	Y	N	N	N	N
Solomon Islands	Y	N	N	Y	Y	N	N	N
Tokelau	Y	N	N	N	N	N	N	N
Tonga	Y	N	N	Y	N	N	N	N
Tuvalu	N	N	N	Y	N	N	N	N
Vanuatu	N	N	N	Y	Y	Y	N	N
Wallis and Futuna	N	Y	N	N	N	N	N	N
**Total number of countries and areas**	**29**	**17**	**6**	**21**	**13**	**14**	**4**	**3**
**Total number of doses ** **administered (millions)**	**433.7**	**101.8**	**97.4**	**68.7**	**18.5**	**10.6**	**1.2**	**0.3**

SAR: Special Administrative Region.

^a^ Includes Sputnik V and Sputnik light.

Adverse events following immunization in the Western Pacific Region

The reporting rates of total AEFIs and serious AEFIs were 130.1 and 5.6 events per 100 000 doses administered, respectively. For both total AEFIs and serious AEFIs, reporting rates in non-PICs and PICs were similar to that for the Western Pacific Region overall (**Table 3**). Rates differed according to vaccine type, with the AstraZeneca vaccine having the highest reporting rate for both total AEFIs and serious AEFIs (**Table 4**).

**Table 3:** Total and serious AEFI reporting rates for COVID-19 vaccines in non-PICs and PICs in the Western Pacific Region, 1 March 2021–31 March 2022

-	Total number of doses administered (millions)	Total AEFIs		Serious AEFIs	
		Number of events reported	Rate (per 100 000 doses administered)	Number of events reported	Rate (per 100 000 doses administered)
**Non-PICs**	730.2	950 031	130.1	40 704	5.6
**PICs**	2.1	2679	129.8	117	5.7
**Total**	732.3	952 710	130.1	40 821	5.6

AEFI: adverse event following immunization; PICs: Pacific island countries and areas.

**Table 4:** Total and serious AEFI reporting rates for COVID-19 vaccines in non-PICs and PICs in the Western Pacific Region by vaccine type,^a^ 1 March 2021–31 March 2022

Vaccine		Total AEFIs n (rate per 100 000 doses)			Serious AEFIs n (rate per 100 000 doses)		
		Total	Non-PICs	PICs	Total	Non-PICs	PICs
*mRNA * *vaccine*	Pfizer- BioNTech	465 901 (107.7)	465 272 (107.7)	629 (80.3)	23 163 (5.9)	23 100 (5.9)	63 (8.1)
	Moderna	191 009 (187.7)	190 894 (188.1)	115 (42.0)	4078 (4.2)	4059 (4.2)	19 (6.9)
*Adenovirus vector-based vaccine*	AstraZeneca	229 331 (333.9)	227 717 (335.3)	1614 (209.1)	7401 (13.5)	7374 (13.6)	27 (3.5)
	Johnson & Johnson	13 621 (128.1)	13 405 (126.9)	216 (324.1)	1135 (10.7)	1128 (10.7)	7 (10.9)
	Gamaleya	875 (71.3)	875 (71.3)	0	37 (3.0)	37 (3.0)	0
*Inactivated vaccine*	Sinopharm	8575 (46.3)	8470 (45.9)	105 (155.4)	107 (0.6)	106 (0.6)	1 (1.5)
	Sinovac	42 670 (43.8)	42 670 (43.8)	0	4885 (5.0)	4885 (5.0)	0
*Protein subunit*	Novavax**^b^**	728 (281.3)	728 (281.3)	0	15 (8.6)	15 (8.6)	0

AEFI: adverse event following immunization; PICs: Pacific island countries and areas.

^a^ In cases where either the numerator (number of events) or the denominator (number of doses administered) was not available separately (i.e. disaggregated by vaccine), or where there were no available data, data were excluded from computation of the AEFI rates.

^b^ The rollout of the Novavax vaccine in the Western Pacific Region started in mid-February 2022, and by the end of March 2022, three countries were using the vaccine (cumulative total number of doses administered = 258 834).

### Adverse events of special interest in the Western Pacific Region

#### Anaphylaxis

**Fig. 1:**
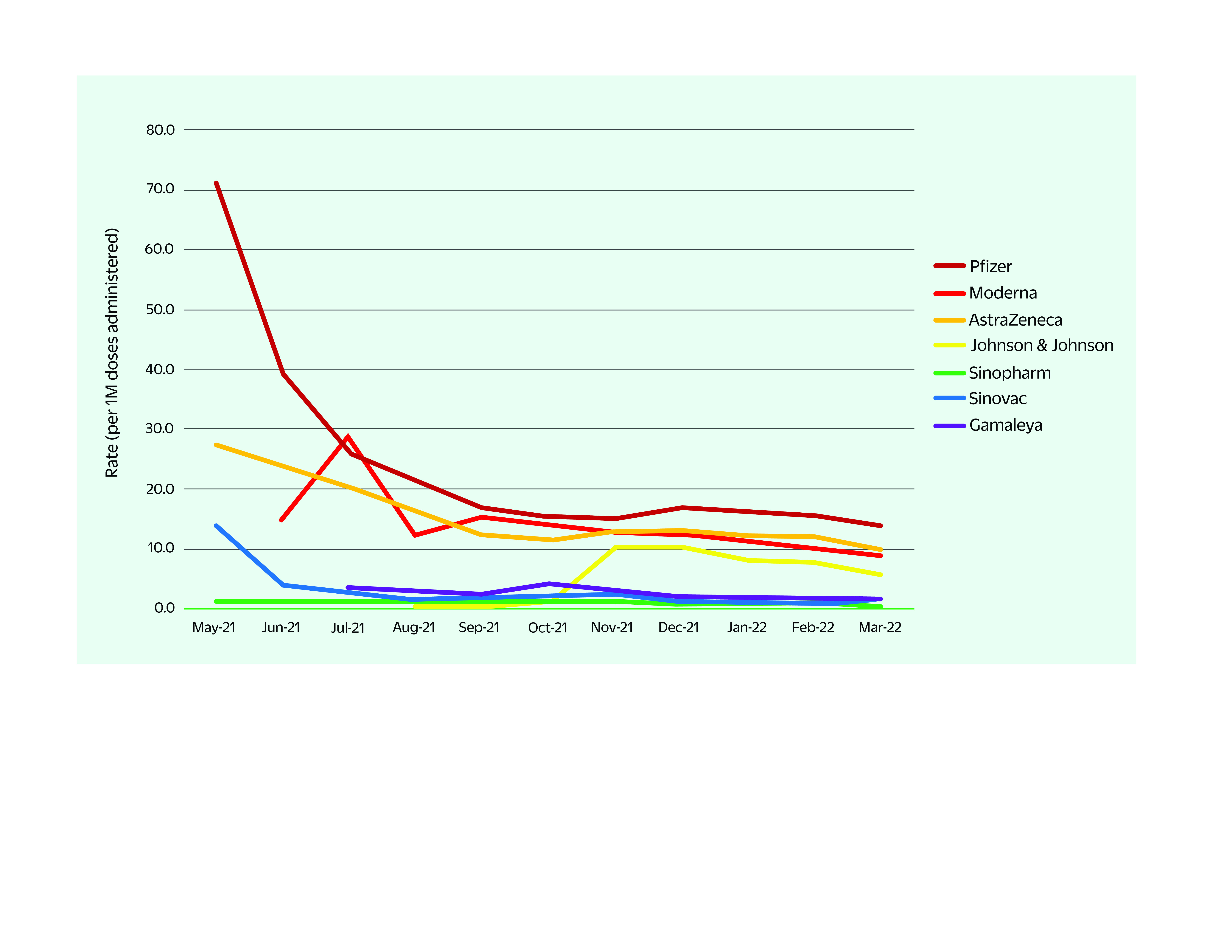
Anaphylaxis reporting rates following administration of COVID-19 vaccinesa in non-PICs, 1 May 2021–31 March 2022

Reporting rates for anaphylaxis by vaccine type ranged from 0.3 (Sinopharm) to 13.7 (Pfizer-BioNTech) cases per 1 million doses administered (**Table 5**). Anaphylaxis reporting rates in non-PICs were higher for most COVID-19 vaccines at the start of the reporting period; these rates then declined and stabilized over the course of the reporting period (**Fig. 1**). The stabilization of anaphylaxis reporting rates coincided with the rise in the number of vaccine doses administered and thus an increase in the size of the denominator.

[**insert** Figure 1]

**Table 5:** Reporting rates^a^ of suspected and/or confirmed anaphylaxis following COVID-19 vaccination in non-PICs and PICs in the Western Pacific Region, 1 March 2021–31 March 2022

-	Non-PICs	PICs	Total
**No. of reporting countries**	14	17	31
**No. of anaphylaxis cases**	7563	3	7566
**COVID-19 vaccine^b^**	**Anaphylaxis reporting rate (cases per 1 million doses administered)**		
Pfizer-BioNTech	13.7	1.3	13.7
Moderna	8.9	0.0	8.9
AstraZeneca	10.0	2.6	9.9
Johnson & Johnson	5.7	0.0	5.6
Gamaleya	1.6	NA	1.6
Sinovac	1.5	NA	1.5
Sinopharm	0.3	0.0	0.3

NA: not applicable; PICs: Pacific island countries and areas.

^a^ In cases where either the numerator (number of events) or the denominator (number of doses administered) was not available separately (i.e. disaggregated by vaccine), or where there were no available data, data were excluded from computation of the AEFI rates.

^b^ Novavax is not included as its rollout only began in February 2022. By the end of March 2022, 14 cases of anaphylaxis had been reported.

#### Thrombosis with thrombocytopenia syndrome

Of the 21 countries and areas that introduced the AstraZeneca vaccine, 12 (10 non-PICs and two PICs) provided data on the number of cases of TTS, of which six (five non-PICs and one PIC) reported no cases. In total, there were 178 suspected and/or confirmed TTS cases following administration of 42.1 million doses of the AstraZeneca vaccine, which equates to a reporting rate of 4.2 cases per 1 million doses administered. Among the six countries that reported TTS cases following the AstraZeneca vaccine, the lowest rate was 0.2 cases per 1 million doses administered and the highest was 17.2 cases per 1 million doses administered.

#### Myocarditis/pericarditis

Seventeen countries and areas (nine non-PICs and eight PICs) used the Pfizer-BioNTech vaccine and reported on myocarditis/pericarditis; of these 17 countries, two non-PICs and six PICs reported zero cases. In the remaining nine countries, there were a total of 5784 reported cases of myocarditis/pericarditis, giving a reporting rate for the Pfizer-BioNTech vaccine in the Western Pacific Region of 15.2 cases per 1 million doses administered. Ten countries and areas (six non-PICs and four PICs) used the Moderna vaccine and also reported on myocarditis/pericarditis. Among this group of countries, half (one non-PIC and all four PICs) reported zero cases; the other five reported a total of 921 cases following the administration of 98.6 million doses. This translates to a reporting rate of myocarditis/pericarditis for the Moderna vaccine of 9.3 cases per  1 million doses administered.

Cases of myocarditis and/or pericarditis were more frequently reported after the second dose of mRNA COVID-19 vaccines in young males aged 12–39 years. Given the clinical and safety importance of myocarditis, particularly among young age groups, a more detailed breakdown of case numbers is provided, by age, sex and dose, for several countries for which such data were available, including Australia, Japan and the Republic of Korea (**Tables 6****–****8**). In all three countries, the highest reported rates of myocarditis were observed in young males following the second dose of the vaccine; reporting rates were higher for the Moderna vaccine than for the Pfizer-BioNTech vaccine. Reported myocarditis rates were generally lower in older adults (i.e. those aged ≥ 30 years); in this age group, rates exhibited little difference between males and females and between the first and second doses for either vaccine.

**Table 6:** Reporting rates of likely myocarditis following Pfizer-BioNTech and Moderna COVID-19 vaccines in Australia, by age and sex (as of 27 March 2022)^a^

Age group^b^ (years)	Pfizer-BioNTech (number of events per 100 000 doses)^c^				Moderna (number of events per 100 000 doses)^c^			
	Both doses		2nd dose		Both doses		2nd dose	
	Male	Female	Male	Female	Male	Female	Male	Female
**12–17**	**7.6**	**1.4**	**12.2**	**2.3**	**10.8**	**3.0**	**20.5**	**5.1**
**18–29**	4.2	1.2	4.5	2.0	8.6	1.1	17.8	2.4
**30–39**	1.6	0.6	2.0	0.7	2.4	0.6	5.1	0
**40–49**	0.7	0.5	1.0	1.0	1.4	0.3	1.7	0
**50–59**	0.4	0.3	0.1	0.3	0.3	0.9	0	2.5
**60–69**	0.1	0.3	0	0.4	0	0.3	0	0
** ≥ 70**	0	0.1	0	0.4	0	0.2	0	0
**Total**	2.0	0.7	3.9	1.1	3.2	0.8	9.6	1.9

^a^ Likely myocarditis includes cases classified as levels 1–3. Level 1 cases are confirmed to be myocarditis based on strong clinical evidence including the patient’s symptoms, and results of tests and imaging indicating a diagnosis of myocarditis. Level 2 cases are probable myocarditis based on a combination of symptoms and routine tests for heart conditions. Level 3 cases are possible myocarditis based on symptoms and a doctor’s report that myocarditis is the most likely diagnosis in the absence of medical tests and investigations. For all cases of suspected myocarditis, where possible, other known causes of the patient’s symptoms or test results are ruled out before cases are classified.

^b^ As of 27 March 2022, no likely cases of myocarditis had been reported in children aged 5–11 years.

^c^ The rate includes cases of myocarditis that occurred after vaccination but may not be vaccine-related. To comply with the Therapeutic Goods Administration’s copyright, the rates are expressed per 100 000 doses administered. To comply with the Therapeutic Goods Administration’s copyright, the rates are expressed per 100 000 doses administered.

*Source:* **Data are reproduced with permission from the Therapeutic Goods Administration, Australian Government.** (*14*)

**Table 8:** Reporting rates of suspected myocarditis following Pfizer-BioNTech and Moderna COVID-19 vaccines in Japan, by age and sex (as of 5 December 2021)

Age group (years)	Pfizer-BioNTech (number of events per 1 million doses)				Moderna (number of events per 1 million doses)			
	Both doses		2nd dose		Both doses		2nd dose	
	Male	Female	Male	Female	Male	Female	Male	Female
**10–14**	13.4	1.5	21.6	1.1	42.3	0	89.8	0
**15–19**	12.9	2.5	21.9	1.7	50.6	1.3	86.5	2.5
**20–24**	8.2	0.6	12.2	0	27.9	1.1	51.9	1.1
**25–29**	6.0	0.9	10.2	1.2	19.7	1.4	34.7	2.9
**30–34**	2.4	0.8	3.0	0	5.9	1.6	10.9	0
**35–39**	1.3	1.5	2.0	0.9	1.5	1.5	2.0	1.6
**40–44**	2.1	0.9	3.8	0.4	3.0	1.5	4.0	1.5
**45–49**	0.8	0.6	0.7	0.6	2.6	2.6	4.4	2.6
**50–54**	0.8	0.9	1.0	0.3	0.5	2.2	1.0	3.0
**55–59**	1.1	0.3	1.1	0.7	1.3	0	2.6	0
**60–64**	0.4	0.8	0.7	1.3	0	0	0	0
**65–69**	0.9	0.4	0.6	0	2.1	2.9	4.3	0
**70–74**	0.4	0.8	0	0.2	0	0	0	0
**75–79**	0.7	0.1	0.4	0	0	0	0	0
** ≥ 80**	1.0	0.9	0.8	0.9	0	0	0	0

Data include Brighton Collaboration level 1–5 cases.

*Source:* **Data are reproduced with permission from the Ministry of Health, Labour and Welfare, Japan.** (*15*)

**Table 7:** Rates of confirmed myocarditis following Pfizer-BioNTech and Moderna COVID-19 vaccines in the Republic of Korea, by age and sex (as of 31 January 2022)

Age group (years)	Pfizer-BioNTech (number of events per 1 million doses)				Moderna (number of events per 1 million doses)			
	1st dose		2nd dose		1st dose		2nd dose	
	Male	Female	Male	Female	Male	Female	Male	Female
**12–17**	12.9	4.3	23.6	3.7	–	–	–	–
**18–19**	14.6	8.2	14.7	8.3	13.9	0.0	47.5	15.6
**20–29**	4.7	4.7	5.4	1.3	10.0	10.4	37.2	4.8
**30–39**	10.4	5.7	2.2	3.3	12.3	9.4	7.8	6.4
**40–49**	2.1	2.5	2.9	2.9	4.3	7.6	3.3	3.9
**50–59**	1.2	4.0	1.9	2.3	1.9	8.6	1.9	0.0
** ≥ 60**	0.0	0.0	0.7	0.4	0.0	0.0	0.0	0.0
**Total**	4.8	3.5	5.0	2.4	6.4	8.4	11.5	3.5

*Source:* **Data are reproduced with permission from the Korea Disease Control and Prevention Agency, WHO Global Advisory Committee on Vaccine Safety meeting, unpublished presentation, January 2022.**

#### Guillain-Barré syndrome

Of the 21 countries and areas in the Western Pacific Region using the AstraZeneca vaccine, 11 (nine non-PICs and two PICs) reported on GBS. Eight of these 21 countries (six non-PICs and two PICs) reported that they had no cases of GBS in the period covered by this study. There were a total of 172 reported suspected and/or confirmed GBS cases in the other 13 countries, suggesting a reporting rate for the AstraZeneca vaccine of 4.1 cases per 1 million doses administered. There was a marked difference in reporting rates between countries, the lowest being 0.93 cases per 1 million doses administered and the highest being 11.59 cases per 1 million doses administered. The Republic of Korea reported two confirmed cases of GBS in people given the Johnson & Johnson vaccine.

## Discussion

This regional analysis summarizes data on AEFIs and AESIs following COVID-19 vaccination as reported by 36 of the 37 countries and areas in the Western Pacific Region during the period of 1 March 2021 to 31 March 2022. The total and serious AEFI reporting rates were used to monitor the functionality of vaccine safety surveillance systems. (*7*)

The total AEFI reporting rate in the Western Pacific Region during the study period was 130.1 cases per  100 000 doses administered; the serious AEFI reporting rate was 5.6 cases per 100 000 doses administered. For both categories of adverse events, total AEFIs and serious AEFIs, reporting rates in non-PICs and PICs were similar, suggesting that all countries and areas in the Western Pacific Region had a basic functional surveillance system for monitoring vaccine safety during the COVID-19 vaccination programme. This is a significant improvement compared with 2018, when only 12 countries met the WHO AEFI reporting rate of 10 cases per 100 000 surviving infants, the indicator recommended by the Global Vaccine Action Plan 2011–2020 for monitoring the functionality of countries’ AEFI surveillance systems. (*16*)

Vaccine safety data have been monitored and shared by WHO with countries in the Region through various platforms; however, the findings need to be interpreted with caution. Across the Western Pacific Region, there was a wide variation in the capacity of countries to detect, diagnose, report, investigate and establish causality of AESIs. Although efforts were taken to ensure completeness and accuracy of the aggregated data provided by countries, it was not possible to verify or validate individual cases of reported AESIs. This limitation is expected when cases of AESIs are reported through passive surveillance systems and because of the large scale of the COVID-19 vaccination rollout, which resulted in a high volume of reports within a relatively short period.

The reported rates of anaphylaxis following COVID-19 vaccination in countries and areas in the Region ranged from 0.3 to 13.7 cases per 1 million doses administered, depending on the type of vaccine. This is in line with the mean anaphylaxis rate of 10.7 cases per 1 million doses administered associated with four COVID-19 vaccines (Moderna, Pfizer-BioNTech, AstraZeneca and Johnson & Johnson) reported by the United States Vaccine Adverse Event Reporting System and the European EudraVigilance, (*17*) and also with anaphylaxis rates for the most commonly administered non-COVID-19 vaccines (which ranged from 1 to 10 cases per 1 million doses administered depending on the vaccine). (*17*) The anaphylaxis reporting rate for all COVID-19 vaccines was ranked fifth compared with non-COVID-19 vaccines. (*17*)

The high reporting rates for anaphylaxis that were observed in the early period of the COVID-19 vaccination programme and at the start of our study period were not inconsistent with reporting rates for non-COVID-19 vaccines used in global immunization programmes. For any new vaccine there is a possibility of higher-than-expected rates for anaphylaxis; however, over time the rates tend to return to the expected range due to the high number of doses being administered. In the Western Pacific Region, as the number of vaccines being administered increased as the COVID-19 immunization programme was rolled out, the reporting rates of anaphylaxis stabilized over time. This is an important observation, and one that provides reassurance of the safety of COVID-19 vaccines.

Reports of TTS following COVID-19 vaccination raised concerns across the Region and globally. In the Western Pacific Region, there were 4.2 reported cases of TTS following immunization with AstraZeneca vaccines per 1 million doses administered (range, 0.2–17.2 cases per 1 million doses administered). According to the WHO interim recommendations for use of the AstraZeneca COVID-19 vaccine and data from the global safety database, TTS reporting rates ranged from 0.2 cases per 1 million doses administered in Asian countries to 17.6 cases per 1 million doses administered in European countries. (*18*) This wide range may be a reflection of country variation in TTS detection and/or reporting capacities, as well as a lack of well defined case definitions of TTS in the early period of the COVID-19 vaccination programme. (*10*) Furthermore, as the diagnosis of TTS requires several tests, including imaging and laboratory tests, countries with fewer clinical specialists such as radiologists and haematologists and where diagnostic facilities are more limited may have reduced capacity to detect and report TTS cases. It is also possible that as TTS appears to be age-specific (more commonly reported in people aged < 50 years), (*19*) the age restrictions for obtaining COVID-19 vaccinations implemented by some countries in 2021 (which tended to favour the older age groups) may have affected the reporting rates.

The global COVID-19 vaccination programme also flagged myocarditis/pericarditis as a potential AESI following administration of COVID-19 mRNA vaccines. In the Western Pacific Region, the reported rate of myocarditis/pericarditis for the Moderna vaccine was 9.3 cases per 1 million doses administered, while for the Pfizer-BioNTech vaccine, the rate was 15.2 cases per 1 million doses administered. This compares with reported rates of 104.5 and 97.7 cases per 1 million doses, respectively (as of 27 March 2022), in Australia; (*20*) 29.7 and 22.7 cases per 1 million doses, respectively, in Canada; (*14*) 26.8 and 15.9 cases per 1 million doses, respectively, in the United Kingdom of Great Britain and Northern Ireland; (*21*) and 7.7 and 5.9 cases per 1 million doses, respectively (as of 24 March 2022), in the Republic of Korea. (*22*) Myocarditis and pericarditis data from countries in the Western Pacific Region (**Tables 6****–****8**) and several other WHO regions have also shown that for both vaccines, reporting rates were highest in young males and higher after the second dose than after the first. (*23*) These data suggest that reporting rates stratified by age and sex would be useful for the monitoring of safety profiles of mRNA COVID-19 vaccines in the future.

Overall, 4.1 cases of GBS were reported for every 1 million doses of the AstraZeneca vaccine that were administered in countries and areas in the Western Pacific Region (range, 0.9–11.6 cases per 1 million doses). This is consistent with the reporting rate published by the European Medical Agency (4.4 cases per 1 million doses administered) (*13*) but lower than that in the United States of America (8.2 cases per 1 million doses administered of the Johnson & Johnson vaccine, as of June 2021). (*24*) In July 2021, WHO reviewed the reports of GBS following administration of adenovirus vector-based vaccines, the AstraZeneca and Johnson & Johnson vaccines, and found no evidence to suggest that use of these vaccines was associated with an increase in GBS case rates. (*13*) The reporting rates of GBS for the AstraZeneca and Johnson & Johnson vaccines are lower than that of the 1976 inactivated influenza vaccine (10 cases per 1 million doses administered) but higher than that for seasonal influenza vaccines (1–2 cases per 1 million doses administered). (*25*) This warrants further monitoring of GBS following COVID-19 vaccines and more studies to properly evaluate the potential association of GBS with COVID-19 vaccines.

The COVID-19 vaccination programme provided the opportunity for countries in the Western Pacific Region to expand and strengthen their vaccine and immunization safety surveillance programmes to provide timely detection, reporting and response to safety events, and to ensure the safety of vaccine recipients. The data included in this report suggest that there were functional vaccine safety surveillance systems throughout the Region. In general, high-income countries and areas have greater capacities for surveillance and response to vaccine and immunization safety events than low- and middle-income countries (LMICs) and PICs, particularly in the case of new AESIs. The vaccine and immunization surveillance capacities of many LMICs, particularly at the subnational level and in PICs, are still limited, particularly in the investigation and causality assessment of AESIs. During the course of the pandemic, WHO has provided technical support to several countries in the form of new guidelines, tools and training of country staff.

It is anticipated that the reporting rates presented in this paper will be useful for evaluating the safety performance of COVID-19 vaccines as part of future programmatic and policy decision-making, particularly if COVID-19 vaccines are to be used in a life-course approach and integrated with routine immunization programmes. However, as most of the data stem from passive or enhanced passive surveillance systems, interpretation of the reported AEFI and AESI rates requires caution, as passive surveillance systems are subject to detection and reporting bias. Furthermore, the effect of confounding factors (e.g. age and sex), which are not accounted for in the reporting of some AESIs, cannot be ruled out.

With uncertainty around the continuation of the COVID-19 pandemic, planning and implementing strategies to build resilience for routine immunization programmes beyond COVID-19 vaccination will remain a challenge for many countries. Vaccine benefits far outweigh the risk of reported serious adverse reactions and serious outcomes of COVID-19. (*26*) Adopting a transparent approach to identifying AESIs helps build public trust and can be part of effective risk communication strategies aimed at preventing vaccine hesitancy, which is often grounded in vaccine safety concerns. Thus, to maintain trust in and demand for regular immunization and improve their management of serious AEFI response, countries should sustain the enhancements to their AEFI surveillance programmes made at the national level during the pandemic and further strengthen subnational capacities in AEFI investigation and causality assessment.
